# Determinants of never-treated status in rural versus urban contexts for individuals with schizophrenia in a population-based study in China

**DOI:** 10.1186/s12888-021-03651-y

**Published:** 2022-02-17

**Authors:** Lawrence H. Yang, Michael R. Phillips, Xianyun Li, Gary Yu, Margaux M. Grivel, Jingxuan Zhang, Qichang Shi, Zhijie Ding, Shutao Pang, Ezra Susser

**Affiliations:** 1 Department of Social and Behavioral Sciences, New York University School of Global Public Health, New York, NY USA; 2 Department of Epidemiology, Mailman School of Public Health, Columbia University, New York, NY USA; 3 Shanghai Mental Health Center, Shanghai Jiao Tong University School of Medicine, 3210 Humin Road, Shanghai, 201108 People’s Republic of China; 4 Department of Psychiatry, Columbia University, New York, NY USA; 5 New York State Psychiatric Institute, New York, NY USA; 6 Beijing Hui Long Guan Hospital, Beijing, China; 7 Rory Meyers College of Nursing, New York University, New York, NY USA; 8 Shandong Provincial Mental Health, Jinan City, Shandong Province China; 9 Tong De Hospital of Zhejiang Province, Hangzhou City, Zhejiang Province China; 10 The 3rd People’s Hospital of Tianshui City, Tianshui, Gansu Provice China; 11 Qingdao Blood Center, Qingdao City, Shandong Province China

**Keywords:** Schizophrenia, Psychiatric services, Epidemiology, Antipsychotics, Healthcare utilization, China

## Abstract

**Background:**

A goal of China’s 2012 National Mental Health Law is to improve access to services and decrease urban versus rural disparities in services. However, pre-reform data is needed for objective evaluation of these reforms’ effectiveness. Accordingly, this study compares the pre-reform utilization of medical services for the treatment of schizophrenia in rural and urban communities in China.

**Methods:**

In a large community-based study in four provinces representing 12% of China’s population conducted from 2001 to 2005, we identified 326 individuals with schizophrenia (78 never treated). Comparing those living in urban (*n* = 86) versus rural (*n* = 240) contexts, we used adjusted Poisson regression models to assess the relationship of ‘never treated’ status with family-level factors (marital status, family income, and number of co-resident family members) and illness severity factors (age of onset, symptom severity and functional impairment).

**Results:**

Despite similar impairments due to symptoms, rural patients were less likely to have received intensive mental health services (i.e., use psychiatric inpatient services), and appeared more likely to be ‘never treated’ or to only have received outpatient care. Among rural patients, only having more than four co-resident family members was independently associated with ‘never-treated’ status (RR = 0.34; 95% CI, 0.12–0.94; *p* = 0.039). Among urban patients, only older age of onset was independently associated with ‘never-treated’ status (RR = 1.06; 95% CI 1.02–1.10, *p* = 0.003).

**Conclusions:**

Identifying differential drivers of service utilization in urban and rural communities is needed before implementing policies to improve the utilization and equity of services and to define metrics of program success.

## Background

Schizophrenia is a severe psychiatric illness that contributed to an estimated 12.66 million disability-adjusted life years (DALYs) lost across 20 million global cases according to the 2017 Global Burden of Disease study [1, 2]. In low- and middle-income countries, schizophrenia is ranked the 3rd leading cause of DALYs, accounting for over 15 million DALYs [3]. In China, relative to common mental disorders (e.g., anxiety, depression), schizophrenia in particular is marked by severe stigmatization [4] and, in rural areas especially, people with schizophrenia are especially vulnerable to high mortality and suicide rates [5] and face increased exposure to violence [6]. A marked burden of disease also extends to caregivers, who often face economic, social and psychological consequences associated with caring for individuals with schizophrenia [6].

To address the impacts of schizophrenia and mental disorders overall, China has invested substantial resources in several major mental health initiatives over the past 15 years to improve the accessibility and quality of mental health services [7]. The China *National Information System for Psychosis* began in two provinces in 2004 to monitor the treatment of community-dwelling individuals with severe mental illnesses; as of 2017 this system was monitoring 5.4 million individuals with psychosis from around the country, making it the largest community-based mental health service network in the world [8]. In 2012, China passed its first national mental health law, with a clear emphasis on expanding community-based services [9]. In 2016, the 2015–2020 National Mental Health Work Plan [10] provided specific numeric targets for achieving the aspirational goals outlined in the 2012 Mental Health Law. However, despite rapid improvement in the overall national access and quality of health services, China has simultaneously seen an increased gap in the access and quality of health services – including mental health services – between the rich eastern provinces and the more rural western provinces [11].

Understanding why these reforms are not decreasing the gap in availability and quality of urban versus rural services in China – a situation that may also be occurring in other rapidly developing low- and middle-income countries (LMIC) – is essential to achieving the United Nations’ Sustainable Development Goal of equity in health services [12]. However, despite conducting a national mental health epidemiological study from 2013 to 2015 [13], there has been no systematic effort to clarify the reasons for the persistent (and increasing) gap in urban versus rural services.

Assessment of the effect of China’s mental health reforms on urban versus rural access to care should be based on a comparison of the situation before and after instituting the reforms. This report provides a pre-reform baseline to which subsequent findings can be compared by assessing factors affecting urban versus rural access to care for schizophrenia – one of the mental disorders most likely to be treated– based on data from a large (*n* = 63,004) representative mental health epidemiological study in China conducted from 2001 to 2005 [14]. The findings support that individuals with psychotic disorders overall are more likely to seek treatment (12%) compared to other diagnostic groups including mood, anxiety, and substance use disorders (4.9, 3.2%. 0.8%, respectively) [14]. Individuals with severe psychotic disorders are even more likely to seek treatment (63.5%) compared to severe mood, anxiety, and substance use disorders (6.7, 8.5, 2.8%) [14]. The authors posit numerous factors that contribute to low treatment seeking in non-psychotic disorders in China including low awareness that the illness is treatable or perceived ineffectiveness of treatment, fear of stigmatization, and lack of access to treatment [14]. Importantly, there are notable differences in treatment rates for schizophrenia between urban and rural locales in China; in rural China, data to date indicates that cases are significantly more likely to be untreated (35.4%) compared to their urban counterparts (17.5%) [15]. Determinants of never being treated within rural settings include demographic factors such as older age [16–18], low education [16, 17], and unemployment [16]; family-related factors include lower rates of marriage [17], having no family caregiver or fewer relatives [16–18], and lower family income [18]. For urban settings, determinants of never being treated include illness factors such as later age of onset and greater severity of illness [19]. Comparisons of determinants of never being treated in rural vs. urban locales for individual with schizophrenia are few in number, and these few existing studies have substantial methodological limitations, including not being nationally or regionally representative [20–22]. Further, the “Hukou”, or internal passport system used in China, likely contributes to rural-urban disparities in treatment given that fewer state-sponsored benefits (including health insurance benefits) are afforded those with rural hukou status compared to those with urban hukou status [23].

We compare treatment status between 86 urban and 240 rural community residents with schizophrenia identified in this study to assess the following hypotheses: 1) urban residents with schizophrenia are more likely to be treated (i.e., with more intensive forms of psychiatric care) than rural residents with schizophrenia [21]; 2) ‘illness-severity factors’ such as age of onset [24] and severity of psychiatric symptoms [25] are more important determinants of treatment status (i.e., receiving psychiatric care) in urban settings (where treatment services are more readily available) than in rural settings; and 3) ‘family-level’ factors [26] such as number of adult co-resident family members, marital status, and family income are more important determinants of treatment status in rural settings (where obtaining treatment depends more heavily on family caregivers and household resources [17];) than in urban settings.

## Methods

Details of the two-stage epidemiological study analyzed in this report are described in a previous publication [14]. In brief, the sampling frame included 113 million individuals (≥18 years), representing 12% of China’s adult population. Multistage stratified random-sampling methods identified 363 sampling (267 rural and 96 urban) sites in four provinces (Shandong, Gansu, Qinghai, and Zhejiang). Random selection of all eligible households (one adult per household) in the sampling sites identified 66,554 adults (age 18 and above).

The first-stage assessment was conducted by trained psychiatric nurses and included the 12-item General Health Questionnaire (GHQ) supplemented by eight items assessing specific risk factors for mental disorders [27, 28]. The 63,004 (94.7%) who completed this first-stage assessment were classified into three strata indicating risk for a mental disorder: a) high-risk (*n* = 10,815; 17%): GHQ score ≥ 4 (on a 12-point scale) or ≥ 1 mental health risk factors; b) moderate-risk (*n* = 10,599; 17%): GHQ score 1–3 and 0 mental health risk factors; or c) low-risk (*n* = 41,590; 66%): GHQ score = 0 and 0 mental health risk factors. Repeated assessment of a random subsample of respondents (*n* = 6717; 10.7%) by blinded interviewers found good inter-rater reliability in this three-level classification of risk of a mental disorder (*kappa* = 0.84).

A total of 17,598 respondents were selected for the second-stage assessment, including all 10,815 high-risk subjects and a random sample of 6783 moderate- or low-risk subjects. Trained attending-level psychiatrists administered the Chinese version of the Structured Clinical Interview for DSM-IV -TR psychiatric diagnoses (SCID-IV) and assessed Global Assessment of Functioning (GAF) [29]. The SCID-IV was completed in 16,577 (94.5%) respondents; repeated assessment of SCID-IV in a random subsample of respondents (*n* = 2579, 15.6%) by blinded interviewers found good interrater reliability for the diagnosis of schizophrenia (*kappa* = 0.94).

At each assessment stage informed consent was obtained from the respondents. Each site’s local institutional review board approved the study.

### Participants in this analysis

Among the 16,577 individuals administered the SCID-IV, 6352 had one or more current (past month) SCID DSM-IV-TR diagnosis, including 327 with schizophrenia, 1 of which was missing information on rural/urban status; hence 326 individuals with schizophrenia total are included in the current analysis. By province, 200 individuals with schizophrenia were from Shandong (64 urban, 136 rural); 83 from Gansu (13 urban, 70 rural); 20 from Qinghai (4 urban, 16 rural); and 23 from Zhejiang (5 urban, 18 rural). Basic demographic characteristics (age, sex, years of formal schooling) and other relevant characteristics (religion [does or does not report any religious affiliation or practices], health insurance status [self-pay vs. insured], current employment status [full- or part-time employment vs. unemployed or retired], and current living status [with family or alone]) of participants were obtained as part of the initial screening interview.

### Classification of urban versus rural residence

Urban areas in China are officially designated as ‘cities’ or ‘towns’ [30]. ‘Cities’ must meet specific criteria, including total population (≥80,000 residents, with > 60,000 non-agricultural), economic development (e.g., ratio of gross domestic product attributable to industry), and infrastructure (e.g., extensiveness of paved road) [30]. ‘Towns’ are seats of county-level governments or centers of mining/industry [30]. More than 95% of our sample lived in their assigned *hukou* district.

### Lifetime treatment status

As part of the screening interview, respondents and their family informants were asked: *“In any time in the past, have you (he/she) ever sought help from the following individuals or institutions for help with psychological or mental problems: 1) an inpatient psychiatric ward; 2) outpatient psychiatric services; 3) a general (non-psychiatric) Western-style medical doctor; 4) a Traditional Chinese Medicine (TCM) doctor; 5) a shaman; 6) friends or colleagues; or 7) relatives?”* Reported sources of help were further evaluated as part of the second-stage diagnostic interview by the attending psychiatrist. We then categorized lifetime treatment status (noting urban vs. rural breakdown) as follows: 1) 78 (urba*n* = 15, rural = 63) ‘never received formal treatment’ included those who reported never using any of these sources of support (*n* = 76; urban = 15, rural = 61), and who had *only* sought help from friends or colleagues (*n* = 1; rural = 1), or relatives (*n* = 1; rural = 1); 2) 176 (urban = 61, rural = 115) ‘any prior psychiatric hospitalization’ were those who had *ever* been admitted to an inpatient psychiatric facility (there were very few psychiatric inpatient settings in general hospitals at the time of the study, so virtually all inpatient services were in specialized psychiatric hospitals); and 3) 72 (urban = 10, rural = 62) ‘only received outpatient treatment’ included those who had not been admitted to an inpatient facility but who had ever received outpatient psychiatric care (*n* = 56; urban = 8, rural = 48), who had never received formal psychiatric care but had ever been treated in a general (western medicine) outpatient setting for psychological problems (*n* = 9; urban = 1, rural = 8), those who had not received psychiatric or general (western-style) medical treatment for psychological problems but had received treatment by a TCM doctor (*n* = 5; rural = 5), and those who had *only* sought help from shamans (*n* = 2; urban = 1, rural = 1). Virtually all individuals who received inpatient or outpatient psychiatric care would have been exposed to standard treatment with antipsychotic medication, and many (though not all) of those who only received treatment from non-psychiatric western or TCM physicians would also have been provided short-term antipsychotic medication. The ‘ever treated’ classification (including the inpatient and outpatient groups) is considered meaningful in China because even brief exposure to antipsychotic medication is associated with an improved course of psychosis when compared with no treatment [31].

### Family-level variables

Variables assessing ‘family-level factors’ including number of co-resident adult family members, current marital status (currently married vs. currently unmarried), and mean annual family income were collected during the initial screening interview. We dichotomized the number of co-resident adults family members variable as < 4 versus > 4 because 4 or more adults in a household implies the presence of non-parental family members from younger generations (e.g., either an adult child or a middle-aged sibling [32]) who may be more inclined to view psychotic symptoms as a treatable mental illness [33].

### Illness severity variables

As part of the SCID-IV examination, the attending psychiatrist determined the age of onset (i.e., age of first occurrence of hallucinations, delusions, or thought disorder) and the GAF scale [29, 34]. Duration of illness was the time interval between the onset of illness and the diagnostic interview. The GAF assesses the worst symptom severity and level of impairment in the prior week on a scale of 0 to 100 (lower scores representing more severe symptoms and impairment [34];). The inter-rater reliability of this measure was good (*ICC* = 0.88).

### Statistical methods

The demographic, family-level, illness-severity, and lifetime treatment status characteristics of patients from urban and rural communities are compared. Statistical significance is computed using Chi-square tests for categorical variables, Mann-Whitney U tests for ranked or non-normal continuous variables, and independent samples t-tests for normally distributed continuous variables.

Treatment patterns differed by urbanicity, so we used separate adjusted Poisson regression models for rural and urban residents with a log-link function and a robust variance estimator [35, 36] to compare ‘family-level factors’ and ‘illness severity factors’ in predicting ‘never treated’ (vs. ‘ever treated’) status. We estimate relative risks of never receiving treatment (with 95% CI) derived from the adjusted Poisson regression models. In cross-sectional studies such as ours, relative risks derived from regression models are more accurately interpreted as “prevalence ratios”, but we retain the term “relative risk” for clarity. The relative risks reported in our models can be interpreted as the likelihood of never receiving treatment if one resides in an urban (or rural) setting.

To construct the adjusted models for urban and rural locales, for each model we first entered all ‘family-level factors’ and ‘illness severity factors’ and subsequently added the other variables one at a time: sex, years of schooling, current employment status, insurance status, province (Shandong, Gansu, Qinghai and Zhejiang), religion, and duration of illness. Due to high multicollinearity between age and age of onset (r[309] = 0.62, *p* < 0.0001), we excluded age and retained age of onset in each model because of its prominence in our conceptualization as an ‘illness severity factor.’ Beyond the family-level and illness-severity factors, none of the additional variables considered significantly affected the prediction of ‘never-treated’ status and, thus, were excluded from both the urban and rural models. Analyses were conducted using SAS Version 9.2 [37].

All tests were two-tailed, and *alpha* was set at *p* < 0.05. Cases with missing data were excluded from the corresponding analyses.

## Results

### Comparison of characteristics and treatment status of urban and rural community residents with schizophrenia

Table 1 shows the comparison of the characteristics of urban versus rural community members with schizophrenia. Illness-severity factors, including age of onset, duration of illness, and GAF score were not significantly different by locale, but several family-level factors were significantly different. The level of education and mean per capita family income were greater in urban patients than in rural patients, while the proportion who have ever married and the proportion who were currently employed were significantly greater in rural patients (probably due to the younger age of marriage in rural areas [38] and the ability of rural patients to continue to participate in manual labor on the family farm [39]). Urban residents were also much more likely to have health insurance (which typically includes coverage for mental health services in China) and to have been admitted to a psychiatric inpatient service (70.9% [61/86] vs. 47.9% [115/240]). Conversely, rural residents appeared more likely to be ‘never treated’ (26.3% [63/240] vs. 17.5% [15/86]) and to only have received outpatient care for their condition (25.8% [62/240] vs. 11.6% [10/86]).

**Table 1:** Comparison of characteristics of urban and rural individuals with schizophrenia from China (2001–2005)

Characteristic	Urban (*N* = 86)		Rural (*N* = 240)		*t* (df)	*p*^*+*^
	n	mean (sd)	n	mean (sd)		
**Age**	86	44.7 (14.4)	240	43.0 (13.0)	*t*(324) = 1.01	0.331
**Years of schooling**	86	8.8 (3.6)	240	5.4 (3.9)	*t*(324) = 7.27	**< 0.001**
**Age of onset**	81	30.8 (11.3)	217	29.9 (10.6)	*t*(296) = 0.63	0.527
**Duration of illness (years)**	81	14.1 (11.7)	217	12.5 (10.5)	*t*(296) = 1.18	0.240
**Global Assessment of Function (GAF) score**	86	45.3 (16.1)	239	43.2 (14.3)	*t*(323) = 1.13	0.261
Characteristic	Urban		Rural		Z	*p*^*+*^
	n	median (IQR)	n	median (IQR)		
**Number co-resident family members**^1^	81	2.0 (2.0–3.0)	222	3.0 (2.0–3.0)	*Z = 1.65*	0.099
Annual per capita family income ($US)*^1^	86	486 (316–759)	238	194 (121–316)	*Z = 8.39*	**< 0.001**
Characteristic	Urban		Rural		*X*^*2*^ (df)	*p*^*+*^
	n/N	%	n/N	%		
**Sex**					*X*^*2*^ (1)=0.59	0.442
Male	45/86	52.3%	114/240	47.5%		
Female	41/86	47.7%	126/240	52.5%		
**Marital Status**					*X*^*2*^ (1)=5.52	**0.019**
Currently unmarried	44/86	51.2%	88/240	36.7%		
Currently married	42/86	48.8%	152/240	63.3%		
**Occupation**					*X*^*2*^ (1)=147.57	**< 0.001**
Employed	19/80	23.8%	210/227	92.5%		
Unemployed	61/80	76.2%	17/227	7.5%		
**Religion**					*X*^*2*^ (1)=3.14	0.076
non-religious	77/86	89.5%	195/240	81.2%		
religious**	9/86	10.5%	45/240	18.8%		
**Has health insurance**					*X*^*2*^ (1)=13.15	**< 0.001**
Yes	44/86	51.2%	67/229	29.3%		
no (self-pay)	42/86	48.8%	162/229	70.7%		
**Prior psychiatric treatment**					*X*^*2*^ (2) = 14.05	**< 0.001**
prior hospitalization	61/86	70.9%	115/240	47.9%		
only outpatient treatment	10/86	11.6%	62/240	25.8%		
never treated^2^	15/86	17.5%	63/240	26.3%		

^+^statistically significant *p*-values are bolded

^1^ indicates comparison of medians using Mann Whitney Test

^2^ includes indiviudals who only sought help from friends, colleagues or relatives

*based on 1 July 2003 exchange rate of 8.236 RMB = 1$US

**includes Catholic, Protestant, Buddhist and Taoist

### Bivariate comparison of characteristics of ‘ever treated’ versus ‘never treated’ patients stratified by rural and urban residence

Given the large differences in the types of treatment received by urban and rural residents with schizophrenia, the factors associated with treatment status were examined separately for the urban and rural subsamples of patients.

#### Rural residence

Compared to ‘ever-treated’ patients with schizophrenia, ‘never-treated’ patients are significantly older, have significantly less education, and have significantly fewer co-resident adult family members (Table 2). However, there were no statistically significant differences in marital status, severity of illness parameters (i.e., age of onset, GAF score) or health insurance status between ‘ever-treated’ and ‘never-treated’ patients.

**Table 2:** Comparison of characteristics of ever- vs. never-treated rural-dwelling individuals with schizophrenia in China (2001–2005)

Characteristic	Ever-treated (*N* = 177)		Never-treated (*N* = 63)		*t* (df)	*p*^*+*^
	n	mean (sd)	n	mean (sd)		
**Age**	177	42.0 (12.6)	63	46.0 (13.8)	*t*(238) = 2.12	**0.035**
**Years of schooling**	177	5.9 (3.7)	63	3.9 (3.9)	*t*(238) = 3.69	**< 0.001**
**Age of onset**	164	29.2 (10.3)	53	32.2 (11.4)	*t*(215) = 1.80	0.073
**Duration of illness (years)**	164	12.4 (10.3)	53	12.7 (11.2)	*t*(215) = 0.19	0.853
**Current Global Assessment of Function (GAF) score**	176	43.5 (14.6)	53	42.6 (13.4)	*t*(237) = 0.45	0.655
Characteristic	Ever-treated		Never-treated		Z	*p*^*+*^
	n	median (IQR)	n	median (IQR)		
**Number co-resident family members**^1^	164	3.0 (2.0–3.0)	58	2.0 (2.0–3.0)	*Z = 2.63*	**0.009**
Annual per capita family income ($US)*^1^	176	209 (106–344)	62	182 (121–283)	*Z = 1.46*	0.147
Characteristic	Ever-treated		Never-treated		*X*^*2*^ (df)	*p*^*+*^
	n/N	%	n/N	%		
**Sex**					*X*^*2*^ (1)=0.82	0.367
male	96/177	54.2%	30/63	47.6%		
female	81/177	45.8%	33/63	52.4%		
**Marital Status**					*X*^*2*^ (1)=0.0009	0.976
never married	65/177	36.7%	23/63	36.5%		
ever married	112/177	63.3%	40/63	63.5%		
**Occupation**					*Fisher’s exact*	0.784
employed	153/165	92.7%	57/62	91.9%		
Unemployed	12/165	7.3%	5/62	8.1%		
**Religion**					*X*^*2*^ (1)=0.20	0.655
non-religious	145/177	81.9%	50/63	81.9%		
religious**	32/177	18.1%	13/63	18.1%		
**Has health insurance**					*X*^*2*^ (1)=0.18	0.675
yes	51/170	30.0%	16/59	27.1%		
no (self-pay)	119/170	70.0%	43/59	72.9%		

^+^statistically significant p-values are bolded

^1^ indicatesomparison of medians using Mann Whitney Test

*based on 1 July 2003 exchange rate of 8.236 RMB = 1$US

**includes Catholic, Protestant, Buddhist and Taoist

#### Urban residence

Compared to ‘ever-treated’ patients, ‘never-treated’ patients have a significantly older mean age of onset (29 vs. 40 years of age) and a corresponding shorter duration of illness (Table 3). In urban locales only, compared to ‘ever-treated’ patients, among ‘never-treated’ patients there was a non-significant increased proportion who reported religious beliefs and, surprisingly, a non-significant increased proportion who reported having health insurance. However, there were no statistically significant differences in GAF score or in the family-level parameters (i.e., family income, marital status, number of co-resident family members) between ‘ever-treated’ and ‘never-treated’ patients.

**Table 3:** Comparison of characteristics of ever- vs. never-treated urban-dwelling individuals with schizophrenia in China (2001–2005)

Characteristic	Ever-treated (*N* = 71)		Never-treated (*N* = 15)		*t* (df)	*p*^*+*^
	n	mean (sd)	n	mean (sd)		
**Age**	71	44.2 (14.1)	15	47.4 (15.7)	*t*(84) = 0.79	0.434
**Years of schooling**	71	9.0 (3.6)	15	8.1 (3.5)	*t*(84) = 0.89	0.374
**Age of onset**	69	29.2 (9.6)	12	40.1 (15.9)	*t* (12)=2.31	**0.039**
**Duration of illness (years)**	69	15.2 (12.1)	12	7.8 (7.0)	*t* (25)=2.99	**0.006**
**Global Assessment of Function (GAF) score**	71	44.5 (14.9)	15	49.4 (21.0)	*t*(84) = 1.08	0.283
Characteristic	Ever-treated		Never-treated		Z	*p*^*+*^
	n	median (IQR)	n	median (IQR)		
**Number co-resident family members**^1^	69	2.0 (2.0–3.0)	12	2.5 (2.0–3.0)	*Z = 0.09*	0.928
Annual per capita family income ($US)*^1^	71	486 (304–801)	15	607 (364–729)	*Z = 0.96*	0.338
Characteristic	Ever-treated		Never-treated		*X*^*2*^ (df)	*p*^*+*^
	n/N	%	n/N	%		
**Sex**					*X*^*2*^ (1)=0.43	0.513
male	35/71	49.3%	6/15	40.0%		
female	36/71	50.7%	9/15	60.0%		
**Marital Status**					*X*^*2*^ (1)=0.15	0.701
never married	37/71	52.1%	7/15	46.7%		
ever married	34/71	47.9%	8/15	53.3%		
**Living status**					*X*^*2*^ (1)=0.0009	1.000
lives with family members	57/71	80.3%	13/15	86.7%		
lives alone	12/71	16.9%	2/15	13.3%		
**Occupation**					*Fisher’s exact*	1.000
employed	16/67	23.9%	3/13	23.1%		
unemployed	51/67	76.1%	10/13	76.9%		
**Religion**					*Fisher’s exact*	0.053
non-religious	66/71	93.0%	11/15	73.3%		
religious**	5/71	7.0%	4/15	26.7%		
**Has health insurance**					*X*^*2*^ (1)=1.75	0.186
yes	34/71	47.9%	10/15	66.7%		
no (self-pay)	37/71	52.1%	5/15	33.3%		

^+^statistically significant p-values are bolded

^1^ indicates comparison of medians using Mann Whitney Test

*based on 1 July 2003 exchange rate of 8.236 RMB = 1$US

**includes Catholic, Protestant, Buddhist and Taoist

### Poisson regression models predicting ‘never-treated’ status stratified by rural and urban residence

#### Rural residence

After forcing the three family-level factors (i.e., number of family members, marital status, and family income) and the two illness-severity factors (i.e., age of onset and GAF score) into the model, the only factor that is significantly associated with lifetime treatment status for individuals with schizophrenia is the number of coresident adult family members (dichotomized as 0 to 3 [*n* = 251] versus 4 or more [*n* = 52]). The adjusted model shown in Table 4 indicates that individuals with schizophrenia living in rural communities who live in households with four or more adult family members were 66% *less* likely to be ‘never-treated’ than those living in households with less than four adult family members. Neither of the illness-severity factors were significantly associated with treatment status.

**Table 4:** Predictors of ‘never-treated’ status of rural and urban individuals with schizophrenia in China

	Rural^1^ (*N* = 200)		Urban^2^ (*N* = 80)	
	Adjusted RR (95% CI)	P	Adjusted RR (95% CI)	P
**Family-level factors**				
Number of co-resident adult family members*	**0.34 (0.12–0.94)**	**0.039**	1.34 (0.16–11.35)	0.789
Never married vs ever married	1.10 (0.61–1.97)	0.752	0.67 (0.20–2.19)	0.504
Mean per capita annual family income	1.00 (1.00–1.00)	0.343	1.00 (1.00–1.00)	0.124
**Illness-severity factors**				
Age of onset	1.02 (1.00–1.04)	0.100	**1.06 (1.02–1.10)**	**0.003**
Current GAF score	0.99 (0.97–1.01)	0.513	0.97 (0.93–1.02)	0.296

*GAF* Global Assessment of Functioning

*dichotomized (0 = 0–3 family members; 1 = 4 or more family members)

^1^*n* = 200 due to missing values

^2^*n* = 80 due to missing values

Missing data were excluded for the stratified Poisson regression models for the urban (n = 80 complete cases out of 86 total cases) and rural (*n* = 200 complete cases out of 240 total cases) sites. The family members variable (*n* = 18 missing cases in the rural sites; *n* = 5 missing cases in the urban sites), mean family income (n = 2 missing cases in the rural sites only), age of onset (*n* = 23 missing cases in the rural sites; n = 5 missing cases in the urban sites) and GAF (n = 1 missing case in the rural sites only) had missing data. Treatment status and marital status did not have any missing data

#### Urban residence

The corresponding analysis in urban residents with schizophrenia found that the only factor associated with lifetime treatment status was age of onset. The adjusted model shown in Table 4 indicates that among individuals with schizophrenia living in urban communities, every additional year in the age of onset of psychosis is associated with a 6% increase in the likelihood of being ‘never-treated’. None of the family-level factors are significantly associated with treatment status.

## Discussion

Given the rapid urbanization of China (19% urban in 1980, 36% in 2000, and 61% in 2019 [40];) and of other LMIC [41], national and regional mental health policies and programs need to be flexible enough to deal with the changing prevalence, care-seeking, treatment, and course of mental disorders that accompany this massive migration from rural to urban communities. This will require detailed monitoring of the prevalence, treatment, and course of mental disorders in both rural and urban settings over time. However, despite the fact that about half of the world’s population currently lives in rural communities [42], studies comparing the status and course of schizophrenia (and other mental disorders) in rural versus urban populations are rare, and the available studies [20–22] often have small sample sizes, are not community-based, and are not nationally or regionally representative. The analyses presented in this paper overcome many of these problems; they are based on the results of one of the largest single-country psychiatric epidemiological studies yet reported [14], which used rigorous diagnostic methods to identify community-dwelling individuals with schizophrenia in representative urban and rural communities in China.

Our three a priori hypotheses about the utilization of health services for the treatment of schizophrenia were supported, but not entirely as expected. 1) Despite similarity in the age of onset, duration of illness, and current severity of the disorder, individuals with schizophrenia from rural communities were less likely than those from urban communities to receive inpatient psychiatric treatment (47.9% vs. 70.9%) and more likely to remain untreated throughout the course of their illness (26.3% vs. 17.5%). 2) Among rural patients, older age, lower levels of education, and fewer co-resident adult family members were associated with failure to receive any treatment. However, only the number of co-resident family members variable remained statistically significant after adjusting for other covariates – other ‘family-level’ factors considered (i.e., marital status and mean family income) were unrelated to care-seeking. 3) Among urban patients, later onset of illness and a shorter duration of illness were associated with failure to receive any treatment; however, only the later age of onset variable remained statistically significant after adjusting for other covariates. For urban patients, the level of education, family income, marital status, number of co-resident family members, and current severity of illness were all unrelated to the lifetime utilization of health services for the treatment of schizophrenia.

Contrary to our expectations, income level, education, and illness severity did not help explain major differences in care-seeking for schizophrenia between urban and rural settings [43, 44]. We hypothesize that the reasons for differential rates and types of care-seeking are due to differences in awareness of and access to mental health services in urban versus rural communities. Differences in awareness and access can change the salience of different factors in the decision about whether or not to seek care for psychotic symptoms manifested by a family member. In rural communities, the presence of more adults in the household would both increase the likelihood that at least one family member would interpret the psychotic symptoms as a psychiatric condition requiring treatment [45–47] and the likelihood that at least one family member would be able to mobilize their social exchange network [48] to figure out how to obtain psychiatric treatment for their ill family member. Another possible explanation is that rural households with more family members have an extra, capable adult family member who is able to accompany the patient to the hospital (often located in urban centers) and to care for the patient while he/she is hospitalized [49]. On the other hand, in urban communities where mental health literacy is higher [33], psychiatric centers are more readily available, and a higher proportion of residents have health insurance that covers psychiatric care [50], onset during early adulthood is more likely to be detected quickly and treated soon after onset, while those with late-onset schizophrenia may not be detected or may be less willing to seek treatment (i.e., due to increased stigma).

### Implications for China’s scale-up of mental health services

These results, along with prior literature in this area [12, 51, 52], provide several potential targets for mental health interventions and several potential measures for assessing the effectiveness of the programs and policies undertaken as part of China’s mental health reforms. Previous reports have highlighted the importance of government-sponsored health insurance [51] and the need to ensure that insurance covers mental health services fully, but unexpectedly, our study did *not* find that health insurance status was associated with seeking care for psychosis in either urban or rural communities. Instead, differences in awareness of psychotic disorders and their appropriate treatments, as outlined above, may play a crucially important role in accessing services in both urban and rural settings. In both urban and rural communities, a key outcome measure is reduction (ideally to zero) in the proportions of individuals with schizophrenia who have never received appropriate treatment for the disorder. Another goal is to decrease the gap in the coverage and quality of services received by urban and rural residents with schizophrenia. A third goal is to decrease the need for inpatient psychiatric treatment (particularly, involuntary inpatient treatment) by increasing the proportion of mental health services provided at the ‘community’ level, including at the outpatient clinics of general hospitals and at community-based health centers. That our study indicates that individuals with schizophrenia in rural locales appear to access outpatient treatment only at higher rates than their counterparts in urban areas could be considered a relative strength because sole use of outpatient psychiatric services averts the isolation and severe marginalization associated with inpatient psychiatric hospitalization in China, which may take place a considerable distance away (i.e., in urban centers) for many rural dwellers. An important caveat to consider, however, is that a greater proportion of psychiatric outpatient service use in rural China could also occur because inpatient services are less widely available in rural areas, or possibly because they are more expensive.

Several policies need to be implemented to achieve these goals. In rural communities, mental health literacy needs to be improved to the point that community members can recognize mental disorders including psychotic disorders [13] and know how to obtain appropriate treatment for these conditions. Stigma – a perennial target for those who promote mental health – is a factor that restricts the utilization of needed mental health services, particularly among older urban residents [53], so ongoing efforts are needed to develop and evaluate innovative, cohort-specific strategies for reducing the stigma and discrimination related to mental disorders. Finally, equity in access to mental health services will not be achieved until quality mental health services are available in all county-level general hospitals around the country.

### Limitations

The study’s cross-sectional design limits causal inference and our ability to observe the changing trajectory of treatment-seeking for schizophrenia over time. We were also unable to ascertain the timing of treatment in relation to illness onset or the duration and type of treatment provided (though it is probable that almost all medically treated individuals received antipsychotic medications). However, previous work has shown that a single treatment contact of persons with schizophrenia in China appears associated with positive effects on the course of illness [31], so it is reasonable to use ‘any lifetime treatment’ versus ‘never treated’ to classify treatment status. Although the reported GAF score considers the current severity of both symptoms and psychosocial impairment, it does not necessarily reflect the most severe level of symptoms over the course of illness, and it does not identify specific symptoms (such as violent behavior [54];) that could precipitate care-seeking.

## Conclusions

These results for China in 2001 to 2005 provide a snapshot of an evolving mental health service network that can be used to assess the effects on mental health services of subsequent rapid urbanization and major changes resulting from important mental health policy initiatives such as China’s 2012 national mental health law [9]. Distinguishing the independent effects of economic development, migration to cities, improvements in public health, and mental health policy initiatives on national mental health will be difficult, and the effects of these factors probably differ in different parts of the country. However, in the absence of a baseline against which current conditions can be compared, this difficult task will be rendered nearly impossible. A clear understanding of the current trajectory of mental health services and of the factors that affect this trajectory is essential to developing, evaluating, and regularly revising effective mental health policies and programs.

These findings may also be relevant for other LMIC that are planning to implement community-based mental health reforms. Such projects need to be preceded by community-specific situation analyses that compare the urban versus rural characteristics and treatment of mental illnesses. The subsequent reforms should be flexible enough to include community- and cohort-specific adaptations that will effectively decrease disparities in the provision of mental health services between urban and rural communities. Such situation analyses should – based on our findings – include assessment of the potential differential effect of family-level factors and illness-severity factors on care-seeking for schizophrenia (and other mental disorders) in urban and rural communities. Clarification of the factors that drive the utilization of services in different subgroups of the population will help facilitate community-specific scale-up efforts to alleviate the global burden of mental disorders [33].

## Data Availability

The datasets used and analyzed during the current study are available from the corresponding author (Dr. Michael Phillips) on reasonable request.

## Abbreviations

LMIC: Low- and middle-income countries
GHQ: General Health Questionnaire
SCID-IV: Structured Clinical Interview for DSM-IV -TR psychiatric diagnoses
GAF: Global Assessment of Functioning
TCM: Traditional Chinese Medicine
